# Prevention of post-surgical abdominal adhesions by a novel biodegradable thermosensitive PECE hydrogel.

**DOI:** 10.1186/1472-6750-10-65

**Published:** 2010-09-09

**Authors:** Bing Yang, ChangYang Gong, ZhiYong Qian, Xia Zhao, ZhengYu Li, XiaoRong Qi, ShengTao Zhou, Qian Zhong, Feng Luo, YuQuan Wei

**Affiliations:** 1 Department of Gynecology and Obstetrics, Second West China Hospital, and State Key Laboratory of Biotherapy and Cancer Center, West China Hospital, West China Medical School, Sichuan University, Chengdu, 610041, China

## Abstract

**Background:**

Post-operative peritoneal adhesions are common and serious complications for modern medicine. We aim to prevent post-surgical adhesions using biodegradable and thermosensitive poly(ethylene glycol)-poly(ε-caprolactone)-poly(ethylene glycol) (PEG-PCL-PEG, PECE) hydrogel. In this work, we investigated the effect of PECE hydrogel on preventing post-surgical abdominal adhesions in mouse and rat models.

**Results:**

The PECE hydrogel in sol state could be transformed into gel in less than 20 s at 37°C. In addition, the PECE hydrogel could be easily adhered to the damaged peritoneal surfaces, and be gradually degraded and absorbed by the body within 14 days along with the healing of peritoneal wounds. A notable efficacy of the PECE hydrogel in preventing peritoneal adhesions was demonstrated in the animal models. In contrast, all untreated animals developed adhesions requiring sharp dissection. Furthermore, no significant histopathological changes were observed in main organs of the hydrogel-treated animals.

**Conclusion:**

Our results suggested that the thermosensitive PECE hydrogel was an effective, safe, and convenient agent on preventing post-surgical intro-abdominal adhesions.

## 1. Background

Post-operative peritoneal adhesions are common and serious complications for patients. The incidence of intra-abdominal adhesions ranges from 67 to 93% after general surgical abdominal procedures and is even up to 97% after gynecologic pelvic opening-operations [1-3]. They can induce a broad range of diseases such as infertility, pain, bowel obstruction, and difficulties experienced during re-operative interventions [4-8].

Peritoneal adhesions generally form in the early post-operative period. For decades, unremitting efforts on the issue are focused on developing products used during laparotomy. Numerous drugs against postoperative adhesion have been tested, and have shown promise in animal models, but few have penetrated into clinical practice [9-11]. An approach to prevent abdominal adhesions is to apply barrier in the form of a polymer solution or synthetic solid membrane, separating the injured regions during peritoneal healing. For polymer solutions such as sodium hyaluronate (HA) and carboxymethylcellulose (CMC), the residence time at the site of administration is relatively brief [9]. For synthetic solid membranes such as oxidized-regenerated cellulose (Interceed) [12], PTFE [13], and HA-CMC (Seprafilm) [14], complete coverage of injured peritoneal surfaces is difficult. In addition, application of those materials in laparoscopy can be cumbersome due to difficulties in handling and fixation to the damaged tissue, which may also compromise their effectiveness as barrier systems [10,11]. The risk of material residues for prolonged periods is also one of the possible negative aspects of solid barriers [15].

For these limitations mentioned above, *in situ *crosslinked hydrogel systems have been explored. These crosslinked hydrogel systems are prepared by chemical modification or inconvenient UV illumination [16-18]. Recently, many crosslinked hydrogels based on hyaluronic acid (HA) have been used to prevent the peritoneal adhesion. However, the relatively long gelation time may be impractical [18]. Hence, it is extremely necessary to find an anti-adhesion agent which is not only effective and convenient for application, but also has a short gelation and retention time.

In our previous work, we have successfully prepared an injectable and thermosensitive poly(ethylene glycol)-poly(ε-caprolactone)-poly(ethylene glycol) (PEG-PCL-PEG, PECE) hydrogel based on PEG and PCL which are biocompatible and have been used in several FDA approved products [19]. PECE hydrogel was proved to be biocompatible, bioabsorbable and thermosensitive, which is a flowing sol at low temperature and forms a non-flowing gel at body temperature [19,20]. Here, the thermosensitive hydrogel system was used for preventing abdominal adhesions, which can be easily applied as a mild viscous sol without spatial restriction, and then quickly changes into a durable and flexible gel when the temperature is increased to body temperature. In addition, adhesiveness to peritoneal wounds, dynamic degradation, and effectiveness in preventing adhesions *in vivo *of the hydrogel were investigated in detail.

## 2. Methods

### 2.1 Materials

Poly(ethylene glycol) methyl ether (MPEG, Mn = 550, Aldrich, USA), ε-caprolactone (ε-CL, Alfa Aesar, USA), hexamethylene diisocyanate (HMDI, Aldrich, USA), stannous octoate (Sn(Oct)_2_, Sigma, USA), All reagents used in this article were analytic reagent (AR) grade, and used as received.

### 2.2 Preparation and characterization of PECE hydrogel

#### 2.2.1 Synthesis and characterization of PECE triblock copolymer

PECE copolymer was synthesized as reported previously [19]. Briefly, PEG-PCL diblock copolymer was prepared by ring opening polymerization of ε-CL initiated by MPEG using Sn(Oct)_2 _as catalyst; PECE triblock copolymer was synthesized by coupling PEG-PCL diblock copolymer using HMDI as coupling agent. Fourier transforms infrared spectroscopy (FTIR, 200SXV Infrared Spectrophotometer, Nicolet, USA) and *^1^H-*nuclear magnetic resonance analysis (*^1^H*-NMR, Varian 400 spectrometer, Varian, USA) were used to characterize the prepared PECE copolymer.

#### 2.2.2 Preparation of PECE hydrogel

PECE triblock copolymer was dissolved in normal saline (NS) at designated temperature at the concentration of 15 wt%, 25 wt%, and 35 wt%, respectively, to form PECE hydrogels, and then these PECE hydrogels were kept at 4°C before use.

#### 2.2.3 Sol-gel-sol phase transition behavior

The sol-gel-sol phase transition behavior of the PECE hydrogel (35 wt%) was investigated by rheometry (AR Rheometer 2000ex, TA Instruments, USA). PECE hydrogel was placed between parallel plates of 40 mm diameter and a gap of 31 μm. The data were collected under a controlled stress (0.5 dyn/cm^2^) and a frequency of 1.0 rad/s. The heating rate was 2°C/min. Gelation times of the PECE hydrogel at 25°C and 37°C was also investigated by rheometry.

### 2.3 *In vivo *application of PECE hydrogel

All animal experiments were approved by the Institutional Animal Care and Use Committee and were in compliance with all regulatory guidelines. Animals were purchased from the Laboratory Animal Center of Sichuan University (Chengdu, China). They were housed at temperature of 20-22°C, relative humidity of 50-60% and 12 h light-dark cycles. Animals were provided with standard laboratory chow and tap water ad libitum. All animals would be in quarantine for a week before treatment.

#### 2.3.1 *In vivo *adhesiveness and degradation behavior of the hydrogel

C57BL/6 mice weighing 20 to 22 g were used to establish the model of surgical adhesion formation [21,22]. We anesthetized mice with a single i.p. injection of 0.15 mL of pentobarbital sodium (10 mg/mL). An anterior midline incision was made through the abdominal wall and peritoneum. The cecum was identified and abraded until visible damage by scrubbing with sterile dry surgical gauze. The damaged cecum was returned to the abdominal cavity, and a 1 × 1 cm apposing parietal peritoneal defect was also created using sterile dry gauze. Then the peritoneum as well as underlying partial muscular layer was excised from the abdominal wall. No attempt of hemostasis or intraperitoneal irrigation was made. The abraded cecum was placed in apposition to the peritoneal wall defect. Then 0.3 mL PECE hydrogel (25 wt%) stored at 4°C was painted on the injured sites and the normal peritoneum around. The incision was finally closed in two layers with 5/0 surgical silk suture. The mice treated with 0.3 mL NS were served as control. Animals were sacrificed by cervical dislocation at predetermined time, and hydrogel adhesiveness to the damaged surfaces or hydrogel residue were investigated. Each animal was evaluated according to the following standard adhesion scoring system[21], which has been widely used in this field: score 0, no adhesion; score 1, one thin filmy adhesion; score 2, definite localized adhesions; score 3, dense multiple visceral adhesions; score 4, dense adhesions extending abdominal wall visceral.

#### 2.3.2 Rat sidewall defect-cecum abrasion model and treatment

Wistar albino female rats weighing 230 to 250 g were used to establish the rat sidewall defect-cecum abrasion model [21,22]. The procedure was similar to the methods mentioned above. A 1 × 2 cm peritoneal defect of the cecum with punctate hemorrhage was made by abrasion, and then a 2 × 2 cm apposing parietal peritoneal defect with punctate hemorrhage was created using scalpel in the right abdominal wall. The two injured surfaces were juxtaposed with 3/0 silk suture in order to induce adhesions for the cecum was too floppy in rats. The incision was closed in two layers with 3/0 silk sutures. Forty eight rats were randomly divided into four groups. Each rat in the first group received the treatment of 1 mL NS. The animals in the second, third, or fourth group received the treatment of 1 mL 15 wt%, 25 wt%, or 35 wt% PECE hydrogel, respectively. Two weeks after the procedure, the animals were sacrificed with an overdose of intravenous sodium pentobarbital and examined for adhesion formation by two observers in a double-blinded manner. Each rat was evaluated according to the above mentioned standard scoring system.

### 2.4 Histologic analysis

Specimens were taken from the damaged caecum, damaged abdominal wall, and adhesion-associated tissues. Then, the specimens were fixed in 4% paraformaldehyde in PBS and were embedded in paraffin. Tissues were sectioned, stained with hematoxylin and eosin (H&E) and then observed for histological assessment by two pathologists in a blinded manner.

### 2.5 Toxicity assessment

To evaluate possible side effects in the PECE hydrogel-treated mice and rats, all the animals were observed after administration of PECE hydrogel, including the general conditions (the activity, energy, hair, feces, behavior pattern, and other clinical signs), body weight, and mortality. After sacrificing, various organs (heart, liver, spleen, lung, kidney, stomach, intestine, brain, and bone marrow, etc.) were harvested and fixed in 4% paraformaldehyde in PBS. These tissues were sectioned, stained with H&E and observed by two pathologists in a blinded manner.

### 2.6 Statistical analysis

Adhesion scores did not always follow a normal distribution. For this, statistical inferences were made using Mann-Whitney U-tests, or Fisher's exact test, using SPSS 10.0 software (Chicago, IL). A *P *value <0.05 on a 2-tailed test was considered statistically significant.

## 3. Results

### 3.1 Characterization of PECE hydrogel

#### 3.1.1 Characterization of PECE copolymer

FTIR and *^1^H*-NMR were used to characterize the chemical structure of the PECE copolymer [19,23]. The M_n _and PEG/PCL block ratio of PECE triblock copolymer calculated from *^1^H*-NMR spectra was 3630 and 1100/2530 respectively. FTIR and *^1^H*-NMR results indicated that the PECE triblock copolymer were prepared successfully.

#### 3.1.2 Temperature-dependent sol-gel-sol transition behavior

**Figure 1:**
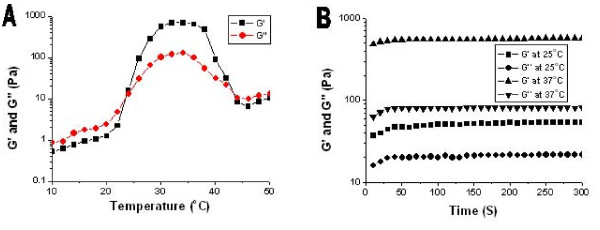
**A: Rheology analysis of PECE-hydrogel (25 wt%) as a function of temperature**. B: Time dependence of storage modulus G' and loss modulus G'' at different temperature.

Gel temperature was defined as the temperature at which storage modulus (G') and loss modulus (G'') were equal. Fig. 1A shows the change in storage modulus (G') and loss modulus (G'') of PECE hydrogel (25 wt%) as a function of temperature. The G' in sol state was less than 10 Pa and increased abruptly by the sol-gel transition at approximately 24°C. When the temperature was 37°C, G' reached about 600 Pa. Then, the dramatic decrease of G' at about 42°C demonstrates the gel-sol transition of PECE hydrogel.

Gelation time was defined as the time when G' became higher than G'' [24]. It reflected the changes in G' and G'' during the gelation process and stood for the gelation speed and gel intensity. Fig. 1A shows that the temperature was 24°C. In Fig. 1B, the gel was formed in less than 50 s at 25°C, which is higher than sol-gel transition temperature. When the temperature increased to 37°C, gel formed in less than 20 s. During the gelation process, G' and G'' increased gradually and eventually reached a plateau, at which G' was significantly higher than G''. This indicated that the PECE hydrogel system displayed a predominantly solid-like behavior at body temperature.

### 3.2 *In vivo *adhesiveness and degradation of PECE hydrogel

The injured surfaces were created on both the cecum and the abdominal wall, and then 0.3 mL of 35 wt% PECE hydrogel was painted on both the wounds and the normal visceral peritoneum around, which quickly transformed into gel state. Animals were sacrificed at predetermined intervals over the next 14 days to assess the adhesiveness and degradation of PECE hydrogel (Table 1).

**Table 1:** Adhesiveness and degradation behavior of PECE hydrogel **(35 wt%) **in the mouse model

Days to dissection	1	3	5	7	14
n	4	4	4	4	4
Presence of residual gel on uninjured viscera	4	4	0	0	0
Presence of residual gel on injured wall	4	4	4	4	0
Presence of residual gel on injured cecum	4	4	4	4	0

On gross examination, the hydrogel adhered to the affected sites and the normal peritoneum was gradually transformed into lubricous viscous liquid due to intestinal peristalsis and peritoneal fluid dilution, and was absorbed by the body within 14 days after the operation. The hydrogel coating the normal peritoneum gradually reduced, and completely disappeared on the 5^th ^day after the operation. Although the hydrogel coating the injured surfaces was also gradually reduced, the residual hydrogel adhering closely to the damaged parietal and visceral surfaces were still observed in each mouse on the 7^th ^day, and completely disappeared within 14 days, which were accompanied with the complete healing of the damaged peritoneal surfaces.

### 3.3 The anti-adhesion effects of PECE hydrogel in mouse model

**Figure 2:**
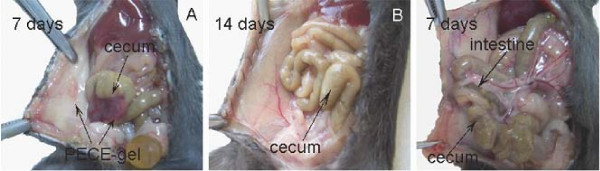
***In vivo *anti-adhesion of PECE gels in mouse model**. A, No apparent adhesion was observed in a PECE gel-treated mouse on the 7^th ^day after the operation, with the gel residue on the damaged surfaces on the abdominal wall and the cecal wall. B, No adhesion was observed in the gel-treated mouse on the 14^th ^day after the operation, the gel was absorbed by the body, with the peritoneal wounds healed. C, Adhesion was observed between the abdominal wall and the cecum as well as the intestine in a saline-treated mouse on the 7^th ^day after the operation, with score 4 adhesion.

The adhesion scores were evaluated on the 7^th ^or 14^th ^day after surgery [22,25,26]. Therefore, we sacrificed all the control mice on the 7^th ^day, in order to compare the intra-abdominal adhesions with those of the hydrogel-treated mice. As shown in Table 2, none of the hydrogel-treated mice developed adhesion (Figs. 2A and 2B), whereas all control mice developed score 4 adhesions (firm adhesion that could only be separated by cutting) (Fig. 2C) (*P *< 0.01, Fisher's exact test). The median adhesion score in the control group was 4, whereas was 0 in the gel-treated group, which was significantly lower (*P *< 0.01, Mann-Whitney *U *test).

**Table 2:** Evaluation of peritoneal adhesions in the mouse model

	Control (n = 5)		PECE (n = 8)	
Adhesion	Frequency	Percentage	Frequency	Percentage
Score 4	5	100	0	0
Score 3	0	0	0	0
Score 2	0	0	0	0
Score 1	0	0	0	0
Score 0	0	0	8	100

### 3.4 The anti-adhesion effects of PECE gels at different concentration in rat model

**Figure 3:**
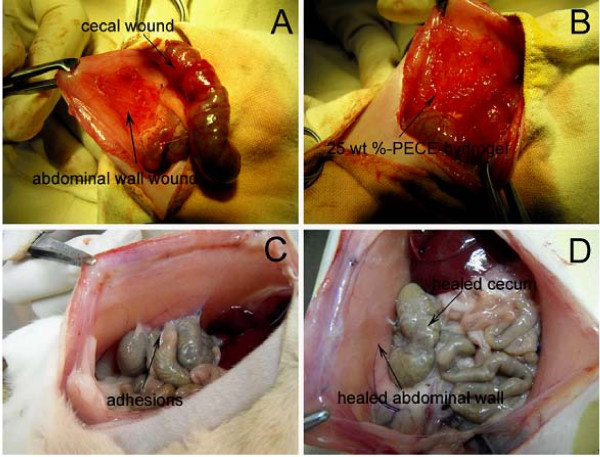
**Prevention of abdominal adhesions in a rat abrasion model**. A, The establishment of rat model of abdominal sidewall defect-cecum abrasion. B, PECE hydrogel applied on the injured abdominal wall and cecum. C, Adhesion was observed in a saline-treated rat. D, No apparent adhesion was observed in a PECE gel-treated rat, with healed abdominal wall and cecum.

Forty-eight rats received laparotomy as described in the methods section (Fig. 3A, B), and were randomly assigned into the following four groups; PECE hydrogel 15 wt%, 25 wt%, 35 wt%, and the control group, respectively.

The adhesion scores for all animals are given in Table 3. The median adhesion scores in the three hydrogel-treated groups were all significantly lower than that in control group (15 wt% *P *< 0.01, 25 wt% *P *< 0.001, 35 wt% *P *< 0.001, Mann-Whitney *U *test). No significant difference was observed between the 25 wt% and the 35 wt% group in the median adhesion scores (*P *> 0.05, Mann-Whitney *U *test), but were both obviously lower than that of the 15 wt% group (*P *< 0.05, Mann-Whitney *U *test). Score 4 adhesion was developed in all control animals (Fig. 3C), but in neither 25 wt% nor 35 wt% group (Fig. 3D) (*P *< 0.001, *P *< 0.001, Fisher's exact test). Two rats in the 25 wt% group developed score 2 adhesion between the omentum and the sutured incision. In 35 wt% group one rat developed score 2 adhesion between the omentum and the incision.

**Table 3:** Evaluation of peritoneal adhesions in the rat model

	Control (n = 12)		PECE (15 wt%) (n = 12)		PECE (25 wt%) (n = 12)		PECE (35 wt%) (n = 12)	
Adhesions	Frequency	Percentage	Frequency	Percentage	Frequency	Percentage	Frequency	Percentage
Score 4	12	100	6	50	0	0	0	0
Score 3	0	0	1	8.3	0	0	0	0
Score 2	0	0	0	0	2	16.7	1	8.3
Score 1	0	0	0	0	0	0	0	0
Score 0	0	0	5	41.7	10	83.3	11	91.7

### 3.5 Histological healing process of hydrogel-treated mice

**Figure 4:**
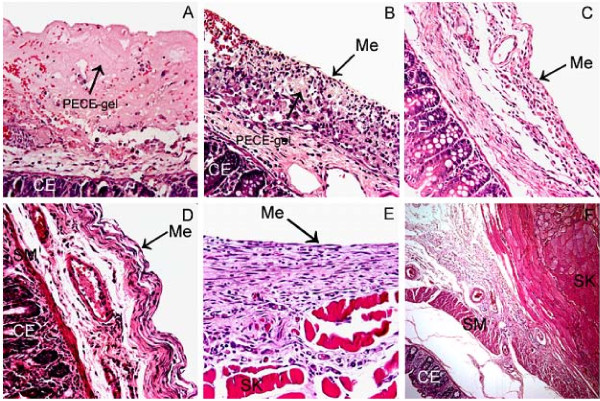
**Histological observations**. A, Adhesion-free cecal wall from a gel-treated mouse 5 days after the surgery (400×). B, Adhesion-free cecal wall from a gel-treated mouse 7 days after the operation. The defect has been remesothelialized (400×). C, Healed cecal wall defect in a gel-treated mouse 14 days after the operation (400×). D, Healed cecal wall and E, healed abdominal wall in a 25 wt% PECE hydrogel-treated rat (400×). F, Cross-section of an adhesion in a saline-treated rat (100×). CE: cecal mucosa; Me: mesothelial cells; SM: visceral smooth muscle; SK: abdominal wall skeletal muscle.

On the 5^th ^day after the operation, a smooth layer of PECE hydrogel was observed on the damaged cecal wall taken from the hydrogel-treated mice. Under light microscope, the injured cecal wall was covered by a layer of pink-stained material, which indicated residual PECE hydrogel stained with eosin, together with erythrocytes, macrophages, and other inflammatory cells (Fig. 4A). The hydrogel-treated defects of the visceral peritoneum began to remesothelialize about 7 days after the treatment, with residual hydrogel, foamy macrophages, macrophages and various fibrosis under the spindle neo-mesothelial cells (Figs. 4B). During the remesothelialization, the hydrogel residues and foamy macrophages under the integral neo-mesothelial cells layer disappeared gradually within the following 7 days, with different degrees of fibrosis (Fig. 4C). The pathological changes of the damaged parietal peritoneum treated with hydrogel were similar to those in the hydrogel-treated visceral peritoneum (data not shown).

Samples of rats taken from the healed abdominal wall and the healed cecal wall showed integral neo-mesothelial cells layers, with various fibrosis (Figs. 4D and 4E). In contrast, pathological examination of samples taken from the adhesion sites in NS-treated rats indicated that the cecal muscular layer was fused to the abdominal wall musculature, with varying thicknesses of intervening inflammation and fibrosis (Fig. 4F).

### 3.6 Toxicity observation

No gross abnormalities were observed in the hydrogel-treated mice and rats. Histological examination of liver, spleen, kidney, heart, pancreas, lung, brain, intestine and bone marrow did not reveal any significant differences between the treated and control groups. No apparent abnormalities were found in the mesothelial cells layers of other abdominal viscera in treated animals.

## 4. Discussion

PECE hydrogel presented in this work was suitable for application in peritoneal cavity. Its physicochemical characteristics and degradation kinetics were appropriate as a promising barrier. It was confirmed by good handling properties during surgery and biological outcomes. The benign nature of the hydrogel was exhibited by its biocompatibility and biodegradability in the mouse and rat models, although long-term safety remains to be demonstrated. Meanswhile, hydrogel with concentration of 25 wt% and 35 wt% showed a notable effect in preventing peritoneal adhesion formation.

Intraperitoneal adhesion is considered to be an inevitable result of surgical trauma to the peritoneum. Trauma initiates an inflammatory response, followed by increase in vascular permeability and release of fibrin-rich exudates and the formation of fibrinous adhesion. If fibrinolysis through the plasminogen-plasmin cascade is not effective enough, fibroblasts invade the fibrinous adhesion. Subsequently, collagen is deposited, leading to the formation of dense fibrous adhesion [9,27,28]. Anti-adhesion barriers act by effectively separating the traumatized peritoneal surfaces during the critical period of adhesion development at 3-5 days after surgery [29-31].

As a physical barrier, the adhesiveness to the traumatized peritoneal surface and biodegradation play key roles in adhesion prevention. Our results showed that the sol state of PECE hydrogel could quickly transform into gel state at body temperature and easily adhere to the damaged peritoneal surfaces. Possible mechanisms might be as follows. Mesothelial cell can secretes surface glycosaminoglycans and a lubricant surfactant similar to type II pneumocytes under normal circumstances [16,32]. However, the injured surface where mesothelial cell layers were stripped facilitates the hydrogels to adhere to the injured locations. In addition, the adhesiveness of hydrogel might be strengthened by the local exudation. We also observed that the hydrogel could be slowly degraded and absorbed during the remesothelialization, and our data revealed that the remesothelialization of the injured parietal and visceral peritoneum treated with PECE hydrogel needed about 7 to 14 days. It is well known that adhesions can be avoided if the integral neo-mesothelial cells layers cover the peritoneal defects. Although the exact effect of PECE hydrogel in anti-adhesion is not clearly understood in a metabolic perspective, it is supposed to be achieved by preventing the apposition of two damaged surfaces during the critical time of adhesion formation and creating favorable conditions for reparative regeneration of mesothelial cells layer[33].

The excellent hydrogel adhesiveness and degradation behavior and preliminary efficacy in anti-adhesion in mouse model guided us to use the rat model which is commonly used in assessment of the efficiency of barrier devices [22]. We chose low, medium and high concentrations of PECE hydrogel to estimate their different efficacy of anti-adhesion and optimize their concentration. The results showed that the effectiveness increased with the increasing concentration of PECE hydrogel within a certain range. Although the ideal concentration of such a system remained unknown yet, it is reasonable to expect that ideal properties could be further optimized by adjusting the concentrations of the polymer and other parameters.

An ideal anti-adhesion barrier should be effective, biocompatible, resorbable, applicable in the laparoscope, and adherent to the traumatized and oozing surfaces. Our results suggested that compared with existing barrier systems, thermosensitive PECE hydrogel has a number of unique physicochemical properties for preventing postoperative adhesions. First, it can be easily extruded through needle and adhere to the affected sites without spatial restriction, which could quickly form a pliable and durable physical barrier at body temperature, without requiring additional cross-linking agents such as initiators or UV illumination. Second, PECE hydrogel and its degradation products can be cleared from the abdominal cavity in a relatively short time after the remesothelialization. Finally, although the damaged peritoneal defects with punctate hemorrhage were left in our models, it was not a handicap for PECE hydrogel (25-35 wt%) to prevent adhesions.

## 5. Conclusions

The PECE hydrogel was highly effective in preventing the formation of postoperative abdominal adhesions. Its thermosensitivity holds great promise compared with existing anti-adhesion barrier systems. It is easy to handle and can be applied with great flexibility, which provides an excellent physical barrier during the critical period of adhesion formation and can be cleared in a short time after the healing process is completed.

## Competing interests

The authors declare that they have no competing interests.

## Authors' contributions

QZY, ZX, WYQ, LF, YB and GCY designed the experiments. And the research funds were supported by WYQ, QZY, ZX and LF. YB, GCY and QXR carried out experiments, analyzed the data, and wrote the manuscript; QZY, ZX, LZY, ZST and ZQ corrected the manuscript. QXR and ZST participated in the animal test of the hydrogels.

All authors approved and read the final manuscript.

## References

[B1] Weibel MA Majno G Peritoneal adhesions and their relation to abdominal surgery. A postmortem study Am J Surg 1973 126 3 345 353 10.1016/S0002-9610(73)80123-0 4580750

[B2] Menzies D Ellis H Intestinal obstruction from adhesions--how big is the problem? Ann R Coll Surg Engl 1990 72 1 60 63 2301905 PMC2499092

[B3] Group OLS Postoperative adhesion development after operative laparoscopy: evaluation at early second-look procedures. Operative Laparoscopy Study Group Fertil Steril 1991 55 4 700 704 1826277

[B4] DeCherney AH diZerega GS Clinical problem of intraperitoneal postsurgical adhesion formation following general surgery and the use of adhesion prevention barriers Surg Clin North Am 1997 77 3 671 688 10.1016/S0039-6109(05)70574-0 9194886

[B5] Ray NF Denton WG Thamer M Henderson SC Perry S Abdominal adhesiolysis: inpatient care and expenditures in the United States in 1994 J Am Coll Surg 1998 186 1 1 9 10.1016/S1072-7515(97)00127-0 9449594

[B6] Coleman MG McLain AD Moran BJ Impact of previous surgery on time taken for incision and division of adhesions during laparotomy Dis Colon Rectum 2000 43 9 1297 1299 10.1007/BF02237441 11005501

[B7] Parker MC Ellis H Moran BJ Thompson JN Wilson MS Menzies D Postoperative adhesions: ten-year follow-up of 12,584 patients undergoing lower abdominal surgery Dis Colon Rectum 2001 44 6 822 829 discussion 829-830 10.1007/BF02234701 11391142

[B8] Fazio VW Cohen Z Fleshman JW van Goor H Bauer JJ Wolff BG Reduction in adhesive small-bowel obstruction by Seprafilm adhesion barrier after intestinal resection Dis Colon Rectum 2006 49 1 1 11 10.1007/s10350-005-0268-5 16320005

[B9] Diamond MP Reduction of de novo postsurgical adhesions by intraoperative precoating with Sepracoat (HAL-C) solution: a prospective, randomized, blinded, placebo-controlled multicenter study. The Sepracoat Adhesion Study Group Fertil Steril 1998 69 6 1067 1074 10.1016/S0015-0282(98)00057-0 9627294

[B10] Liakakos T Thomakos N Fine PM Dervenis C Young RL Peritoneal adhesions: etiology, pathophysiology, and clinical significance. Recent advances in prevention and management Dig Surg 2001 18 4 260 273 10.1159/000050149 11528133

[B11] Attard JA MacLean AR Adhesive small bowel obstruction: epidemiology, biology and prevention Can J Surg 2007 50 4 291 300 17897517 PMC2386166

[B12] Prevention of postsurgical adhesions by INTERCEED(TC7), an absorbable adhesion barrier: a prospective randomized multicenter clinical study. INTERCEED(TC7) Adhesion Barrier Study Group Fertil Steril 1989 51 6 933 938 2524407

[B13] Haney AF Doty E Murine peritoneal injury and de novo adhesion formation caused by oxidized-regenerated cellulose (Interceed [TC7]) but not expanded polytetrafluoroethylene (Gore-Tex Surgical Membrane) Fertil Steril 1992 57 1 202 208 1730318 10.1016/s0015-0282(16)54802-x

[B14] Becker JM Dayton MT Fazio VW Beck DE Stryker SJ Wexner SD Prevention of postoperative abdominal adhesions by a sodium hyaluronate-based bioresorbable membrane: a prospective, randomized, double-blind multicenter study J Am Coll Surg 1996 183 4 297 306 8843257

[B15] Wallwiener M Brucker S Hierlemann H Brochhausen C Solomayer E Wallwiener C Innovative barriers for peritoneal adhesion prevention: liquid or solid? A rat uterine horn model Fertil Steril 2006 86 4 Suppl 1266 1276 10.1016/j.fertnstert.2006.05.023 17008150

[B16] Beavis J Harwood JL Coles GA Williams JD Synthesis of phospholipids by human peritoneal mesothelial cells Perit Dial Int 1994 14 4 348 355 7827184

[B17] Osada H Takahashi K Fujii TK Tsunoda I Satoh K The effect of cross-linked hyaluronate hydrogel on the reduction of post-surgical adhesion reformation in rabbits J Int Med Res 1999 27 5 233 241 10689629 10.1177/030006059902700503

[B18] Liu Y Li H Shu XZ Gray SD Prestwich GD Crosslinked hyaluronan hydrogels containing mitomycin C reduce postoperative abdominal adhesions Fertil Steril 2005 83 Suppl 1 1275 1283 10.1016/j.fertnstert.2004.09.038 15831302

[B19] Gong C Shi S Dong P Kan B Gou M Wang X Synthesis and characterization of PEG-PCL-PEG thermosensitive hydrogel Int J Pharm 2009 365 1-2 89 99 10.1016/j.ijpharm.2008.08.027 18793709

[B20] Gong CY Wu QJ Dong PW Shi S Fu SZ Guo G Acute toxicity evaluation of biodegradable in situ gel-forming controlled drug delivery system based on thermosensitive PEG-PCL-PEG hydrogel J Biomed Mater Res B Appl Biomater 2009 91 1 26 36 19365823 10.1002/jbm.b.31370

[B21] Ersoy E Ozturk V Yazgan A Ozdogan M Gundogdu H Comparison of the two types of bioresorbable barriers to prevent intra-abdominal adhesions in rats J Gastrointest Surg 2009 13 2 282 286 10.1007/s11605-008-0678-5 18777122

[B22] Ersoy E Ozturk V Yazgan A Ozdogan M Gundogdu H Effect of polylactic acid film barrier on intra-abdominal adhesion formation J Surg Res 2008 147 1 148 152 10.1016/j.jss.2007.09.005 18262551

[B23] Gong CY Dong PW Shi S Fu SZ Yang JL Guo G Thermosensitive PEG-PCL-PEG hydrogel controlled drug delivery system: Sol-gel-sol transition and in vitro drug release study J Pharm Sci 2009 10.1002/jps.21694 19189419

[B24] Wei CZ Hou CL Gu QS Jiang LX Zhu B Sheng AL A thermosensitive chitosan-based hydrogel barrier for post-operative adhesions' prevention Biomaterials 2009 30 29 5534 5540 10.1016/j.biomaterials.2009.05.084 19647868

[B25] Kim SY Kim HJ Lee KE Han SS Sohn YS Jeong B Reverse Thermal Gelling PEG-PTMC Diblock Copolymer Aqueous Solution Macromolecules 2007 40 15 5519 5525 10.1021/ma070190z

[B26] Kosaka H Yoshimoto T Yoshimoto T Fujimoto J Nakanishi K Interferon-gamma is a therapeutic target molecule for prevention of postoperative adhesion formation Nat Med 2008 14 4 437 441 10.1038/nm1733 18345012

[B27] De Iaco PA Stefanetti M Pressato D Piana S Dona M Pavesio A A novel hyaluronan-based gel in laparoscopic adhesion prevention: preclinical evaluation in an animal model Fertil Steril 1998 69 2 318 323 10.1016/S0015-0282(98)00496-8 9496348

[B28] Ustun C Yanik FF Kocak I Canbaz MA Cayli R Effects of Ringer's lactate, medroxyprogesterone acetate, gonadotropin-releasing hormone analogue and its diluent on the prevention of postsurgical adhesion formation in rat models Gynecol Obstet Invest 1998 46 3 202 205 10.1159/000010034 9736805

[B29] Burns JW Skinner K Colt J Sheidlin A Bronson R Yaacobi Y Prevention of tissue injury and postsurgical adhesions by precoating tissues with hyaluronic acid solutions J Surg Res 1995 59 6 644 652 10.1006/jsre.1995.1218 8538160

[B30] Rodgers KE Johns DB Girgis W Campeau J diZerega GS Reduction of adhesion formation with hyaluronic acid after peritoneal surgery in rabbits Fertil Steril 1997 67 3 553 558 10.1016/S0015-0282(97)80085-4 9091346

[B31] Reijnen MM Skrabut EM Postma VA Burns JW van Goor H Polyanionic polysaccharides reduce intra-abdominal adhesion and abscess formation in a rat peritonitis model J Surg Res 2001 101 2 248 253 10.1006/jsre.2001.6288 11735283

[B32] Mutsaers SE Mesothelial cells: their structure, function and role in serosal repair Respirology 2002 7 3 171 191 10.1046/j.1440-1843.2002.00404.x 12153683

[B33] Nishioka Y Miyazaki M Abe K Furusu A Regeneration of Peritoneal Mesothelium in a Rat Model of Peritoneal Fibrosis Renal Failure 2008 30 97 105 10.1080/08860220701741619 18197550

